# *Brassica napus* Mediator Subunit16 Induces BnMED25- and BnWRKY33-Activated Defense Signaling to Confer *Sclerotinia sclerotiorum* Resistance

**DOI:** 10.3389/fpls.2021.663536

**Published:** 2021-08-19

**Authors:** Huizhen Hu, Yiwei Tang, Jian Wu, Feizhi Chen, Yidan Yang, Xuancheng Pan, Xiang Dong, Xianda Jin, Sheng Liu, Xuezhu Du

**Affiliations:** ^1^State Key Laboratory of Biocatalysis and Enzyme Engineering, Hubei Collaborative Innovation Center for Green Transformation of Bio-Resources, Hubei Key Laboratory of Industrial Biotechnology, College of Life Sciences, Hubei University, Wuhan, China; ^2^Southwest Research Center for Landscape Architecture Engineering, State Forestry and Grassland Administration, Yunnan Province Engineering Research Center for Functional Flower Resources and Industrialization, College of Landscape Architecture and Horticulture Sciences, Southwest Forestry University, Kunming, China; ^3^Key Laboratory of Plant Functional Genomics of the Ministry of Education, Yangzhou University, Yangzhou, China; ^4^Key Laboratory of Biology and Genetic Improvement of Oil Crops, Ministry of Agriculture and Rural Affairs, Wuhan, China

**Keywords:** BnMED16, BnMED25, BnWRKY33, *Brassica napus*, defense signaling, sclerotinia stem rot

## Abstract

The plant mediator is a highly conserved protein complex that interacts with transcription factors (TFs) and RNA polymerase II (RNAP II) to relay regulatory information during transcription. Plant immune response is one of the biological processes that is orchestrated by this regulatory mechanism. *Brassica napu*s, an important oil crop, is severely attacked by a devastating disease Sclerotinia stem rot. Here, we explored broad-spectrum disease resistant roles of *B. napus* mediator subunit 16 (BnMED16) and its host defense mechanism against fugal pathogen *Sclerotinia sclerotiorum*. We found that *BnMED16* expression was significantly increased by *S. sclerotiorum* infection, and its homologous overexpression resulted in rapid and comprehensive defense responses from the beginning to the end. This affected signal transduction with multiple channels including pathogen recognition, intracellular Ca^2+^ concentration, reactive oxygen species (ROS) accumulation and clearance, and activation of mitogen-activated protein kinase (MAPK) signaling cascades initially. Subsequently, pathogen-/defense-related genes and hormone-responsive pathways were highly activated, which resulted in enhanced cell wall and secretion of defense proteases. Furthermore, the biochemical analysis showed that BnMED16 interacts with BnMED25 and BnWRKY33. Additionally, BnMED25 also interacts with TFs BnMYC2, BnCOI1, and BnEIN3 of the JA/ET signal transduction pathway. Taken together, we proposed a hypothetical model that BnMED16 confers *S. sclerotiorum* resistance by enhancing BnMED25-mediated JA/ET defense pathways and BnWRKY33-activated defense signaling in *B. napus*. The *BnMED16* overexpressing lines with enhanced broad-spectrum disease resistance could be useful for breeding *Sclerotinia*-resistant oilseed rape varieties, as well as serving as basis for further strategy development in resistance breeding.

## Introduction

Oilseed rape (*Brassica napus* L.) is a major oil crop in the world. It is prone to a devastating disease sclerotinia stem rot (SSR), which is caused by *Sclerotinia sclerotiorum* (Lib.) de Bary, a necrotrophic ascomycete (Bolton et al., 2006). This fungus has a broad host range on at least 408 known plant species of 278 genera in 75 families (Boland and Hall, 1994), causing serious crop losses worldwide (Derbyshire and Denton-Giles, 2016). To cultivate resistant varieties is considered to be the most economical and effective way to control SSR, but the highly immune germplasm resources are quite limited in *B. napus* and its related species. To date, the molecular mechanism of SSR resistance, controlled by numerous of quantitative loci, still remains elusive in rapeseed varieties (Wu et al., 2016; Wu et al., 2019).

When plants subjected to pathogens, transcriptional reprogramming signals were produced to shift plant normal growth to disease resistance before basal resistance is activated, and folds changes of the transcriptional signals would determine the resistance to disease (Maleck et al., 2000; Katagiri, 2004; Wu et al., 2016). In this process, the mediator complex (MED) links TFs and RNA polymerase II (RNAP II), and helps the TFs to target on gene promoters (Kidd et al., 2011; Moore et al., 2011). The mediator is a conserved complex consists of four modules, includes head, middle, tail, and cyclin dependent kinase (CDK) (Chadick and Asturias, 2005). The head and tail modules interact with RNAPII and specific TFs respectively, the middle module transmits signals from tail to head, and the CDK module usually acts as a transcriptional suppressor (Casamassimi and Napoli, 2007; Guo et al., 2020). Since MEDs constitute the basis of transcriptional activation or inhibition in specific signal pathways, they are involved in various vital biology activities includes plant growth and development, response to biotic and abiotic stresses, and a variety of intracellular life activities (Kidd et al., 2011).

The plant MED members were initially identified in *Arabidopsis thaliana* (Bäckström et al., 2007) and subsequently in other species (Mathur et al., 2011; Ren et al., 2020). In *Arabidopsis*, the MED contains 27 conserved subunits and 6 specific subunits (Bäckström et al., 2007; Mathur et al., 2011), many of which play pivotal roles in plant disease resistance. For instance, *Arabidopsis MED14* and *MED15* mutants showed inhibited NAD-induced PR1 expression *in vitro* and remarkably reduced resistance to salicylic acid (SA)-triggered immunity against biotrophic pathogens *Pseudomonas syringae* (Canet et al., 2012; Zhang et al., 2013). The *Arabidopsis* MED19a were degraded by oomycete pathogen *Hyaloperonospora arabidopsidis* effector HaRxL44, and significantly reduces plants resistance responses (Caillaud et al., 2013). AtMED8, 12, 13, 16, 21, 25, and CDK8 have been found to regulate the disease resistance against necrotrophic pathogens and activate the jasmonic acid/ethylene (JA/ET) signal pathway, especially for the *Atmed25* mutant, it was susceptible to necrotrophic fungus *Alternaria brassicicola* and *Botrytis cinerea* by significantly reduced JA/ET-mediated immunity against necrotrophic pathogens (Dhawan et al., 2009; Kidd et al., 2009; Zhang et al., 2012; Zhu et al., 2014; Zhai and Li, 2019). Notably, among 14 *Arabidopsis MED* mutants, *med16 (sfr6)* mutant was most susceptible to *S. sclerotiorum*, suggests that AtMED16 is a key positive factor fight against *S. sclerotiorum* (Wang et al., 2015). Besides, AtMED16 was related to AtWRKY33 and mediated WRKY33-activated defense signaling (Wang et al., 2015). In addition, studies have shown that AtMED16 can interact with AtMED25 to regulate iron homeostasis and participate in ABA response (Yang et al., 2014; Guo et al., 2020). It has also been reported that MED16 may be a signaling component in the gap between the transcription coactivator NON-EXPRESSOR OF PATHOGENESIS-RELATED GENES1 (NPR1) signaling node and the general transcription machinery to positively regulate SA-mediated systemic acquired resistance and JA/ET-induced defense pathways (Zhang et al., 2012). These results have led to speculation that the plant conservative MED16 may regulate resistance to *S. sclerotiorum* by mediating signaling pathways activated by multiple TFs. Although increasing evidence has been discovered to support the disease resistance of MEDs in *Arabidopsis*, the molecular mechanisms underlying their resistance is still in its infancy, especially in oilseed rape.

In this study, we identified BnMED16 as a positive central regulator of basal resistance against *S. sclerotiorum* in *B. napus*. We demonstrated that overexpressing *BnMED16* promoted the accumulation of ROS at the early stage and maintained the balance of its accumulation and clearance at the later stage of *S. sclerotiorum* infection. It also increased the expression of pathogen-related (PR) genes, enhanced the plant cell wall reinforcement and cell wall-mediated resistance by lignification and pectins deposition, and induced plant endogenous hormone synthesis and transduction. We further discovered that BnMED16 interacted with BnMED25 and BnWRKY33, while BnMED25 also associates with JA/ET TFs MYC2, CORONA TINE INSENSITIVE 1 (COI1), and ETHYLENE INSENSITIVE3 (EIN3). Taken together, our results indicated that BnMED16 is a key regulator of both BnMED25-mediated JA/ET defense pathways and the BnWRKY33-activated defense signaling in *B. napus*.

## Materials and Methods

### Generation of Transgenic *B. napus*

To generate *BnMED16* overexpressing plants, full-length cDNA of *BnMED16* (*BnaA09g20140D*), an orthologous gene with the highest homology to *AtMED16* (*At4g04920*; Wang et al., 2015) among the BnMED16 isoforms of *B. napus*, was cloned and driven by double CaMV 35S promoter in the binary vector pCAMIBA2301-1300 plasmid for constitutive overexpression. The construct pCAMIBA2301-1300-D35S:*BnMED16* was then transferred into *Agrobacterium tumefaciens* GV3101 and was subsequently used to transform into the susceptible *B. napus* (Westar) by Agrobacterium-mediated transformation (Jonoubi et al., 2005). Untransformed control plants and transgenic lines were grown in growth chambers at 22 ± 2°C under a photoperiod of 16 h light/8 h dark, with a relative humidity of 60%. T0 transgenic seedlings were identified by amplifying *kanamycin* gene and confirmed by qRT-PCR (Real-time quantitative reverse transcription polymerase chain reaction). More than three kanamycin-resistant lines (independent transformation events) were selected as homozygous. Phenotypic characterization and inoculation analyses were performed using T4 homozygous transgenic lines. The information of primers used in this study was listed in Supplementary Table 1.

### Nucleic Acid Isolation and qRT-PCR Analysis

Total genomic DNA was extracted from fresh leaves of wild-type (WT, Westar) and transformed plants using the cetyltrimethylammonium bromide (CTAB) method (Arseneau et al., 2017). Total RNA was isolated using an Eastep^TM^ Super Total RNA Extraction Kit (Promega, Madison, WI, United States), and the first-strand cDNA was synthesized from 1 μg of total RNA in 20 μL reactions using HiScript II Q RT SuperMix for qPCR (Vazyme, Nanjing, China) and oligo-dT(18)-MN primers following the manufacturer’s instructions. qRT-PCR was performed using SYBR^®^ Green Realtime PCR Master Mix-Plus (Takara, Tokyo, Japan) under the following conditions: polymerase activation for 30 s at 95°C, followed by 40 cycles of 15 s at 95°C, 15 s at 60°C and 25 s at 72°C. The *B. napus Actin7* (Gene ID: 106418315) gene was used as a reference for internal control and the gene expression was normalized against the reference gene of *BnActin7*. All of the primers used in these assays are listed in Supplementary Table 1 and the assays were carried out for three biological replicates.

### Pathogen Infection

The *S. sclerotiorum* (Lib.) de Bary isolate SS-1 was maintained and cultured on potato dextrose agar (PDA) medium (213400; Becton–Dickinson) for about 36 h. The uniform agar disk with fungal hyphae was (4 mm in diameter) placed on the detached leaf surface of 6-week-old *B. napus* plants or agar disk with fungal hyphae was (6 mm in diameter) placed on the 50 cm high internode of live stems surface of *B. napus* plants at flowering stage. One rosette leaf/stem per plant was inoculated for basal resistance assessment and the eighth leaf of each plant was removed for detached leaf inoculation with *S. sclerotiorum* in a greenhouse. Ten biological replicates were performed for this experiment. During inoculation, leaves were kept in a growth tray with a transparent cover to maintain high humidity. At 0, 6, 12, 24, 36, 48, and 72 h post-inoculation (hpi), images were taken of inoculated leaves and lesion sizes were measured using the ImageJ 1.32j software^1^.

### RNA-Seq and Data Analysis

The fresh leaves of WT and transgenic plants were collected before (0 hpi) and after (6 hpi) *S. sclerotiorum* inoculation *in vitro* and the samples (three biological replications) were subjected to RNA extraction, mRNA library construction, and RNA sequencing (MGISEQ2000 sequencing platform, BGI Co., Ltd., Shenzhen, China). To identify genes corresponding to reads from each sample library, the reads were aligned to the *B. napus* reference genome^2^ and the *S. sclerotiorum* genome^3^ using TopHat. Gene expression levels were estimated based on fragments per kilobase of exon per million fragments mapped (FPKM), which were used to draw heatmaps for the differentially expressed genes (DEGs) identified with DEGseq based on the criteria *q*-value <0.005 and | log^2^ (FPKM-transgenic/FPKM-WT)| >1 between transgenic and WT lines. For gene ontology (GO) term annotations, all *B. napus* genes were searched against the National Center for Biotechnology Information (NCBI) non-redundant (Nr) protein database using GOSeq with corrected *P*-value < 0.05.

### Hydrogen Peroxide Detection in Inoculated Leaves

The accumulation of H_2_O_2_ in leaves of WT and transgenic plants before (0 hpi) and after (6 hpi) *S. sclerotiorum* inoculation were observed using 3′-3-diaminobenzidine (DAB) staining. Leaves inoculated with *S. sclerotiorum*, and then the uninfected parts around the inoculated site of the leaves were cut into the same size and stained with DAB solution as previously described (Wang et al., 2018). Leaves were then washed in deionized water and treated with 95% (v/v) ethanol to remove chlorophyll. Images were taken under a stereoscopic microscope (VT1000S, Leica, Germany).

The fresh leaves (0.1 g) of WT and transgenic plants before (0 hpi) and after (6 hpi) *S. sclerotiorum* inoculation were collected and extraction by 80% acetone at 4°C, then the supernatants (hydrogen peroxide extraction) were quantitatively analyzed by H_2_O_2_ Quantitative Assay Kit (water-compatible; Rongbai, Shanghai, China) for the quantitative analysis of H_2_O_2_ content.

### Enzyme Activity Determination of β-1-3-Glucanase and Chitinase

The fresh leaves (0.5 g) of WT and transgenic plants before (0 hpi) and after (6 hpi) *S. sclerotiorum* inoculation were collected and extraction by phosphate buffer at 4°C overnight, then the supernatants were quantitatively analyzed by Plant β-1,3 glucanase ELISA Kit and Plant chitinase ELISA Kit (Rongbai, Shanghai, China) for the enzyme activity determination of β-1-3-glucanase and chitinase, respectively. At least three biological replications were performed. LSD test was used for statistic comparisons (*P* < 0.01).

### Yeast Two-Hybrid (Point-to-Point) Assay

The full-length coding sequence of BnMED16 (BnaA09g20140D) and BnMED25 (BnaA09g28640D) was amplified from *B. napus* cDNAs and cloned into the yeast two-hybrid bait vector pGBKT7 (with the Gal4 binding domain) and transformed the resulting plasmid pGBKT7-BnMED16 (BnMED16-BD) and pGBKT7-BnMED25 (BnMED25-BD) into the yeast (*Saccharomyces cerevisiae*) Y2HGold strain, and then grown on the SD-Trp plate to select positive colonies. The full-length coding sequence of BnMYC2 (BnaC05g28450D), BnERF1 (BnaA01g23940D), BnORA59 (BnaC08g44670D), BnEIN3 (BnaA05g20160D), BnCOI1 (BnaA03g56600D), BnWRKY15 (BnaA04g13570D), BnWRKY75 (BnaC09g44020D), BnMED25 (BnaA09g28640D), BnMED14 (BnaAnng10600D), and BnWRKY33 (BnaC04g06800D) was amplified from *B. napus* cDNAs and cloned into the yeast two-hybrid the prey vector pGADT7 (with the Gal4 activation domain) and transformed the resulting plasmids into the yeast strain Y187, and then grown on the SD-Leu plate to select positive colonies. The above two types of positive colonies (positive colonies in Y187 and Y2HGold strains) were co-cultured for mating in the SD-Leu-Trp liquid medium for 22–24 h at 30°C with 50–100 rpm. After dilution, the co-culture suspensions were dropped (5 μL per drop) onto the solid nutrient-deficient mediums (SD-Leu-Trp, SD-Leu-Trp-His, and SD-Ade-His-Leu-Trp). The resulting agar plate was incubated at 30°C and observed for yeast growth. Alleles are mainly selected based on the most differentially expressed genes in RNA-seq. All of the primers used in these assays are listed in Supplementary Table 1 and the assays were carried out for three biological replicates.

### Bimolecular Fluorescence Complementation (BiFC) Assay

To generate the BiFC constructs, the full-length of BnMED16 (BnaA09g20140D) and BnMED25 (BnaA09g28640D) were amplified with primer pairs BnMED16-nYFP-F/R or BnMED25-nYFP-F/R and inserted into a linearized pS1301-35s-Myc-nYFP vector which was digested with *Xba*I and *Kpn*I using an in-fusion enzyme to obtain the nYFP-BnMED16 and nYFP-BnMED25. The full-length of BnMED25 (BnaA09g28640D), BnWRKY33 (BnaC04g06800D), BnMYC2 (BnaC05g28450D), BnCOI1 (BnaA03g56600D), and BnEIN3 (BnaA05g20160D) were amplified with primer pairs BnMED25-cYFP-F/R, BnMEDWRKY33-cYFP-F/R, BnMYC2-cYFP-F/R, BnCOI1-cYFP-F/R or BnEIN3-cYFP-F/R and inserted into a linearized pS1301-35s-HA-cYFP vector which was digested with *Xba*I and *Kpn*I using an in-fusion enzyme to obtain the cYFP-BnMED25, cYFP-BnWRKY33, cYFP-BnMYC2, cYFP-BnCOI1, and cYFP-BnEIN3. Then nYFP-BnMED16 and cCFP-BnMED25, nYFP-BnMED16 and cCFP-BnWRKY33, nYFP-BnMED25 and cCFP-BnMYC2, nYFP-BnMED25 and cCFP-BnCOI1, nYFP-BnMED25 and cCFP-BnEIN3, nYFP-MED16 and cCFP, nYFP-MED25 and cCFP, cCFP-BnWRK33 and nYFP company with nuclear marker (ELONGATED HYPOCOTYL 5, AT5G11260) (Zhao et al., 2019) strain (mCherry-AtHY5,498 nm) were co-infiltrated into the leaves of *Nicotiana benthamiana* via the *A. tumefaciens* strain GV3101, respectively. Fluorescence signals in leaf epidermal cells were observed using a confocal microscope (Leica sp8). The primers used in this study are listed in Supplementary Table 1 and the assays were performed for three biological replicates.

## Results

### Mediator Subunits Response to *S. sclerotiorum* in *B. napus*

**FIGURE 1:**
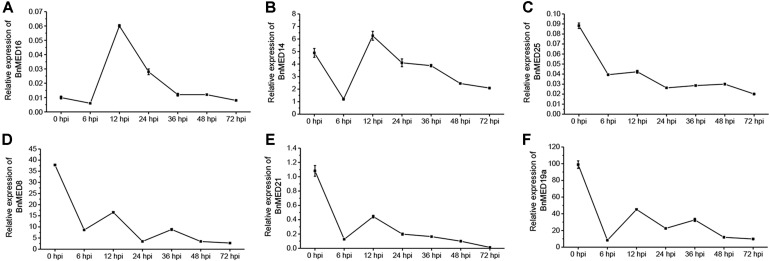
Expression pattern of mediator subunits in *Brassica napus* leaves with the treatment of *Sclerotinia sclerotiorum* at different time points after inoculation. RT-QPCR analyses of *MED16* (*BnaA09g20140D*) **(A)**, *MED14* (*BnaAnng10600D*) **(B)**, *MED25* (*BnaA09g28640D*) **(C)**, *MED8* (*BnaA09g53670D*) **(D)**, *MED21* (*BnaA09g20250D*) **(E)**, and *MED19a* (*BnaC09g44480D*) **(F)** in leaves at seedling stage of wild-type (WT, Westar) inoculated with *S. sclerotiorum*. *BnACTIN7* was used as the internal control; data are means ± SD of three biological replicates; hpi, hours post-inoculation.

Previously, studies have shown that *Arabidopsis* mediator subunits MED14, MED15, MED16, and MED19a showed resistance against biotrophic and hemibiotrophic pathogens (Canet et al., 2012; Zhang et al., 2012; Caillaud et al., 2013), whereas MED8, MED12, MED13, MED14, MED16, MED21, MED25, and CDK8 exhibited resistance to necrotrophic pathogens (Dhawan et al., 2009; Kidd et al., 2009; Zhang et al., 2012; Zhu et al., 2014; Wang et al., 2015). We thus speculated that some certain BnMEDs confer the resistance to *S. sclerotiorum* in *B. napus*. To screen out MEDs involved in regulating the SSR defense response in *B. napus*, we analyzed the expression pattern of *BnMEDs*, each gene with the highest homology to the corresponding *Arabidopsis AtMEDs*, in response to *S. sclerotiorum* at time points after inoculation of *B. napus* WT leaves (Figure 1). Interestingly, we found the transcript levels of *BnMED16* were significantly increased at 12 and 24 h post-inoculation (hpi) with *S. sclerotiorum* by five and twofold compared to that of 0 hpi (Figure 1A), whereas the expression level of *BnMED14* at 12 hpi was only comparable to that of 0 hpi (Figure 1B). However, the transcript levels of other mediator genes such as *BnMED25* (Figure 1C), *BnMED8* (Figure 1D), *BnMED21* (Figure 1E), and *BnMED19a* (Figure 1F) at all time points were remarkably lower than that of 0 hpi. It has been shown that *AtMED16*, the *Abrabidopsis* ortholog of *BnMED16* mediates basal resistance to *S. sclerotiorum* and mutants of *AtMED16* were notably more susceptible to *S. sclerotiorum* than mutants of 13 other mediator subunits in *Arabidopsis* (Wang et al., 2015). We thus speculate that BnMED16 (BnaA09g20140D) is a key positive regulator factor against *S. sclerotiorum* in *B. napus*.

### Overexpression of *BnMED16* Enhanced Resistance to *S. sclerotiorum* in *B. napus*

**FIGURE 2:**
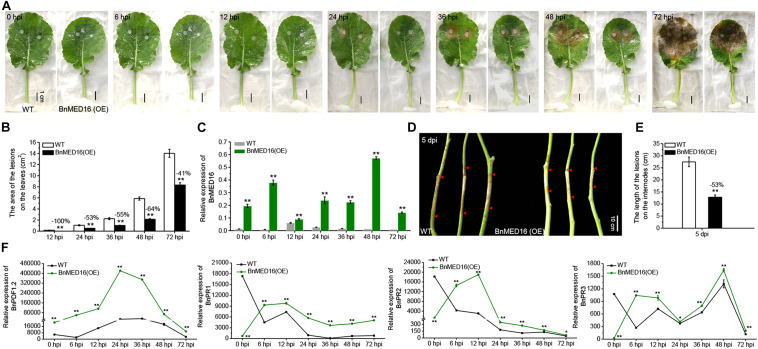
Overexpression of *BnMED16* conferred resistance to *S. sclerotiorum* in transgenic rapeseed plants. **(A)** T4 homozygous BnMED16(OE) lines and WT inoculated with 6 mm diameter agar plugs of *S. sclerotiorum* hyphae at 22°C. Images were taken at 0, 6, 12, 24, 36, 48, and 72 hpi. BnMED16(OE) was the transgenic plants that overexpressed *BnMED16* genes in *B. napus*. Bars, 1 cm. **(B)** Lesion areas in BnMED16(OE) and WT leaves at different time points after inoculation as shown in **(A)**. **(C)** Expression pattern of *BnMED16* in BnMED16(OE) and WT leaves at different time points after inoculation as shown in **(A)**. Data are means (±SD) of three biological replicates. **(D)** Images of BnMED16(OE) and WT stem inoculated with *S. sclerotiorum* after 5 days. dpi, days post-inoculation. Bars, 10 cm. **(E)** Lesion lengths in BnMED16(OE) and WT stem after inoculation as shown in **(D)**. **(F)** Expression pattern of *BnPDF1.2* (*BnaC02g23620D*), *BnPR1* (*BnaC01g04530D*), *BnPR2* (*BnaA01g17540D*), and *BnPR3* (*BnaA05g26640D*) in BnMED16(OE) and WT leaves at different time points after inoculation as shown in **(A)**. *BnActin7* was used as the internal control. The data are the mean ± SD of three independent biological replicates; *n* ≥ 10 leaves **(B)** and *n* = 10 stems **(E)** were measured in each replicate and the asterisks denote statistical significance at *P* < 0.01 (**) or *P* < 0.05 (*) between the WT and BnMED16(OE) lines at each time point by Student’s *t*-tests. Percentage changes relative to the WT are indicated.

To verify the resistance effects of BnMED16 on *S. sclerotiorum*, we generated a *BnMED16* constitutively overexpressing line in a susceptible *B. napus* background (WT; Westar). qRT-PCR analysis of T_0_ independent transgenic lines revealed that the transcript levels of *BnMED16* were significantly increased in line 1 (1#), followed by line 18 (18#), line 3 (3#), line 10 (10#), line 22 (22#), and line 19 (19#) compared to the WT (Supplementary Figure 1), subsequently the transgenic lines (1#, 18#, and 3#) were selected and propagated to homozygous states individually. We found that there was no significantly difference in plant seedling growth between homozygous transgenic lines and WT (Supplementary Figure 2). To test the resistance to *S. sclerotiorum*, detached leaves of T_4_ seedlings were inoculated, pictures or samples were taken, and samples were harvested at 0, 6, 12, 24, 36, 48, and 72 hpi time points (Figure 2A). As compared to the WT, the *BnMED16* overexpressing lines [BnMED16(OE)] were obviously more resistant to infection at 12 hpi, which showed a significant reduction in lesion size (Figure 2B). Then, we analyzed the expression pattern of *BnMED16* in BnMED16(OE) and WT seedling leaves at time points. The result showed that the transcript level of *BnMED16* gene was remarkably increased at all time points compared to inoculated untransformed control leaves with two distinct peaks at 6 and 48 hpi (*P* < 0.01; Figure 2C), suggesting that *BnMED16* expression pattern was positively related to the plant resistance to *S. sclerotiorum* and the response to SSR was faster and stronger in transgenic lines. At the flowering stage, the primary stems of BnMED16(OE) lines and WT were inoculated with isolated *S. sclerotiorum* using hyphal agar plugs. After 5 days, all transgenic individuals showed smaller lesions compared to WT (Figures 2D,E), consistent with the results of leaves inoculated *in vitro* (Figures 2A,B). Together, these data indicate that overexpressing *BnMED16* enhanced rapeseed resistance of SSR both at the seedling and flowering stages under controlled disease stress conditions.

To further evaluate the effect of *BnMED16* on basal defense genes, we analyzed the expression patterns of *PLANT DEFENSIN1.2 (BnPDF1.2)*, *BnPR1*, β*-1-3-glucanase* (*BnPR2*) and *chitinase* (*BnPR3*) genes in BnMED16(OE) and WT seedling leaves with time course. The results displayed that the transcript level of *BnPDF1.2* gene dramatically increased at all time points in BnMED16(OE) leaves compared to WT with a distinct peak at 24 hpi, whereas *BnPR1*, *BnPR2*, and *BnPR3* genes were much higher expressed in BnMED16(OE) leaves than that of WT after 6 hpi (*P* < 0.01; Figure 2F). Furthermore, the enzyme activities of β-1-3-glucanase and chitinase in WT and BnMED16 (OE) leaves at 0 and 6 hpi were analyzed, the results were also consistent with the transcriptional analysis (Supplementary Figure 3). These results suggesting that the increased expression of *BnMED16* enhances the plant basal defense genes level, thus improving the resistance of SSR in *B. napus*.

### Overexpression of *BnMED16* Activates Basal Resistance Against *S. sclerotiorum*

**FIGURE 3:**
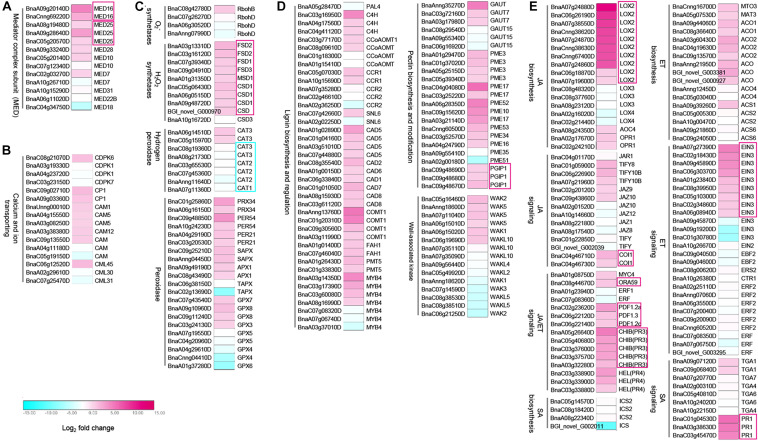
Differentially expressed genes (DEGs) between *B. napus* transgenic and WT lines in response to *S. sclerotiorum* infection. Heatmap of DEGs related to *S. sclerotiorum* infection *B. napus* seedling leaves of mediator genes **(A)**, Ca^2+^-mediated signal transduction related genes **(B)**, ROS related genes **(C)**, plant cell wall-related genes **(D)**, and JA/ET synthesis and signal pathway regulated genes **(E)**. DEGs were considered statistically significant if *q*-value < 0.005 and | log_2_-fold change| > 1. The log_2_-fold change is indicated according to the scale bar. The color from cyan to magenta represents the DEGs from down-regulated to up-regulated. The intensity of the color indicates the multiple of change for DEGs, with a darker color meaning higher fold change. The common up-regulated DEGs are highlighted by red boxes. Details of the genes are given in Supplementary Dataset 1.

Since the mediator complex plays an important role in transcriptome reprogramming process to initiate the transcription of defense-related genes (Kidd et al., 2011; Moore et al., 2011), and our data showed that *BnMED16* overexpressing plants responded to SSR resistance faster and stronger (Figure 2), we speculate that constitutive expression of *BnMED16* might promote the activation of numerous defense-related genes at the early stage of *S. sclerotiorum* infection. To investigate how overexpression of *BnMED16* influences transcriptome after infection, we performed RNA-seq of 6 hpi (the early stage of *S. sclerotiorum* infection) WT and BnMED16(OE) seedling leaves (Supplementary Figure 4). We obtained 14182 differentially expressed genes (DEGs) in total (Supplementary Figure 4A), and they were analyzed through KEGG pathway classifications and gene ontology (GO) term annotations (Supplementary Figures 4B,C) to elucidate the possible resistance pathways. As a result, a large number of DEGs were found related to plant immunity. Mediator subunits *MED25*, *MED28*, *MED10*, and *MED7* including *MED16* were highly expressed in BnMED16(OE) lines (Figure 3A and Supplementary Dataset 1); as an early event of the innate immune system, Ca^2+^-mediated signal transduction related genes, *CDPK-related kinase 6* (*CDPK6*), *calmodulin* (*CAM*) and *calcium-binding* (*CML*) genes, were all significantly increased (Figure 3B and Supplementary Dataset 1); meanwhile the ROS related genes like *H_2_O_2_ synthetases* (*FSD1*, *FSD2*, *FSD3*, *MSD1*, and *CSD1*), *catalase* (*CAT3*) and *peroxidases* (*PRX34*, *PER54*, *PER21*, *APX1*, *GPX7*, and *GPX8*) were also induced in *BnMED16* transgenic plants (Figure 3C and Supplementary Dataset 1); others like mitogen-activated protein (MAP) kinases pathway (Supplementary Figure 5C and Supplementary Dataset 1) and receptor-like protein kinases (RLKs) genes (Supplementary Figure 5D and Supplementary Dataset 1) were also greatly affected by the enhanced expression of *BnMED16*.

Subsequently, genes related to response event of the immune system were also discovered to be differentially expressed in MED16 constitutive overexpression lines. In the plant cell wall reconstruction pathway, the prominent enhanced expression occurs in the majority of lignin synthesis genes (*C4H*, *CCoAOMT*, *CCR1*, *CAD5*, *CAD2*, *CAD6*, *CAD1*, *CAD7*, *CAD8*, *COMT1*, *FAH1*, and *PMT5*), and pectin synthesis or modification genes (*GAUT7*, *GAUT15*, *PME3*, *PME17*, *PME52*, *PME10*, *PME53*, *PME34*, *PME16*, *PME35*, *PME51*, and *PGIP1*). Meanwhile, the expression of oligogalacturonides (OGs) receptor wall-associated kinase (WAK) family genes, a kind of PRRs connects immune responses to necrotrophic pathogens by inducing cell wall-mediated resistance (Li et al., 2009; Brutus et al., 2010), were significantly up-regulated or down-regulated (Figure 3D and Supplementary Dataset 1). Besides, the genes with significantly improved expression levels in the BnMED16(OE) line also include the JA biosynthesis genes (*LOX2*, *AOC4*, and *OPR4*), ET biosynthesis genes (*MTO3*, *MAT3*, *ACO1*, and *ACO2*), JA/ET signaling genes (*MYC4*, *ORA59*, *PDF1.2a*, *PDF1.2c*, *PDF1.3*, *PR3*, *PR4*, *EIN3*, *TIFY8*, *TIFY108*, *JAZ9*, *JAZ10*, and *COI1*), and SA signaling genes (*TGA1* and *PR1*). This indicates that JA/ET synthesis and signal pathway regulated genes dominate SSR resistance in *B. napus* (Figure 3E and Supplementary Dataset 1). We also found that almost all camalexin synthesis genes like *Glutathione transferases* (*GST*), *cytochrome P450* (*CYP79B2*, *CYP71A13*, *CYP71B7*, and *CYP71B28*) and *PHYTOALEXIN DEFICIENT 4* (*PAD4*) were induced dramatically (Supplementary Figure 5B and Supplementary Dataset 1). To validate the data obtained from the RNA-seq, we analyzed the expression patterns of nine genes (*MED25*, *JAZ1*, *MYC2*, *COI1*, *EIN3*, *ORA59*, *ERF1*, *CYP71B7*, and *PGIP1*) in BnMED16(OE) and WT seedling leaves at 0, 6, 12, 24, 36, 48, and 72 hpi. The results showed that the expression levels of these genes were basically consistent with that of RNA-seq at 6 hpi, and expression levels of these genes were significantly boosted during *S. sclerotiorum* infection (Supplementary Figure 6). Taken together, these data suggest the BnMED16 was a key factor of basal resistance against the necrotrophic fungal pathogen *S. sclerotiorum*.

### BnMED16 Effectively Regulate the Balance Between Production and Scavenging of ROS With *S. sclerotiorum* Infection

Reactive oxygen species, a second messenger, regulates the innate plant immune system to enhance defense against disease by uncertain mechanisms (Foyer and Noctor, 2011; Baxter et al., 2014).

**FIGURE 4:**
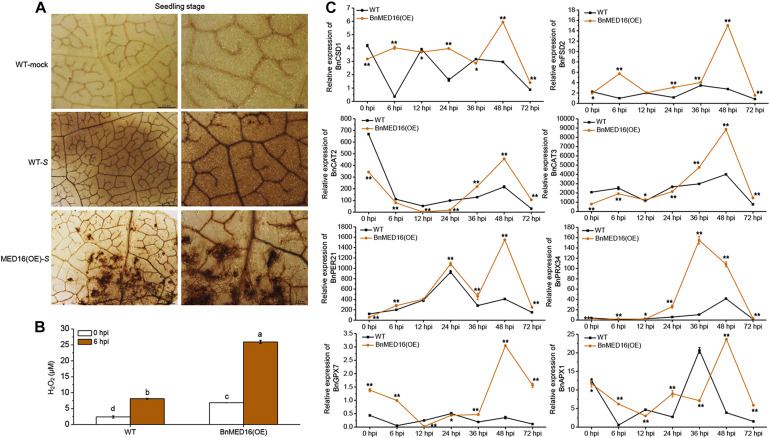
H_2_O_2_ accumulation and the expression levels of ROS-associated genes in response to *S. sclerotiorum* inoculation in BnMED16(OE) and WT of rapeseed. **(A)** Detection of H_2_O_2_ accumulation by DAB staining in *B. napus* leaves at the seedling stage after *S. sclerotiorum* inoculation. The images show leaves at 6 hpi with either mock solution of *S. sclerotiorum* (S). Three biological replicates were used in this experiment; *n* ≥ 10 leaves were measured in each replicate. Bars, 0.5 mm (the first column) and 0.1 mm (the second column). **(B)** Determination of H_2_O_2_ content of seedling leaves after inoculation as shown in **(A)**. The data are the mean ± SD of three independent biological replicates. Least-significant difference (LSD) tests were used for multiple comparisons. Different letters above bars indicate that the means differ according to ANOVA and LSD tests (*P* < 0.01). **(C)** Expression pattern of *BnCSD1* (*BnaA06g05150D*), *BnFSD2* (*BnaA03g13310D*), *BnCAT2* (*BnaA03g53180D*), *BnCAT3* (*BnaA08g21730D*), *BnPER21* (*BnaC03g20530D*), *BnPRX34* (*BnaA01g20660D*), *BnGPX7* (*BnaA03g51760D*), and *BnAPX1* (*BnaA09g49190D*) in BnMED16(OE) and WT seedling leaves at different time points after inoculation with *S. sclerotiorum*. *BnActin7* was used as the internal control; Data are means ± SD of three biological replicates and the asterisks denote statistical significance at *P* < 0.01 (**) or *P* < 0.05 (*) between the WT and BnMED16(OE) lines at each time point by Student’s *t*-tests.

Our RNA-seq data revealed that numerous ROS related genes were differently expressed in BnMED16(OE) and WT plants after *S. sclerotiorum* inoculation (Figure 3C). We thus studied the effects of *BnMED16* overexpression on the accumulation of ROS during the whole infection process. The BnMED16(OE) seedling leaves inoculated with *S. sclerotiorum* after 6 h were more strongly stained by diaminobenzene (DAB; mainly staining for H_2_O_2_-derived production) than that of WT (Figure 4A), and the quantitative analysis of H_2_O_2_ content showed the similar results as DAB staining (Figure 4B), indicated that H_2_O_2_ accumulation in *BnMED16*-overexpressing leaves was strongly induced at the early stage of infection. Interestingly, we found that H_2_O_2_ synthetases genes such as *BnCSD1* and *BnFSD2* increased significantly at 6, 24, and 48 hpi in BnMED16(OE), the expression pattern exhibited a wavy rise or decline with a highest peak at 48 hpi; whereas the expression of catalytic genes like *BnCAT2* and *BnCAT3*, involved in the degradation of H_2_O_2_, was much lower expressed in BnMED16(OE) leaves at 0, 6, 12, and 24 hpi, and they increased dramatically after 36 hpi with a peak at 48 hpi. After *S. sclerotiorum* inoculation, the expression of peroxidases such as *BnPER21* and *BnPRX34* also increased notably with a similar expression pattern in both the transgenic and WT plants, but more significantly in the *BnMED16* transgenic plants with the peak at 36 or 48 hpi. For more intricacy cases of other peroxidases, such as *BnGPX7* and *BnAPX1*, were still highly expressed in BnMED16(OE) lines with a peak at 48 hpi (Figure 4C). Taken together, these results indicated that overexpression of *BnMED16* could enhance the SSR resistance in *B. napus* by promoted faster ROS accumulation at early stage, and controlled ROS content in plants more effectively at late stage when subjected to *S. sclerotiorum.*

### BnMED16 Is Physically Associated With BnMED25

**FIGURE 5:**
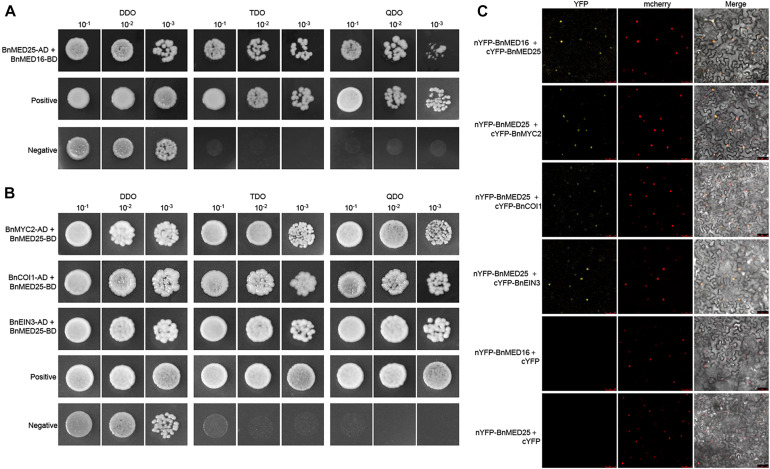
Physical association between *B. napus* mediator and JA/ET-regulated proteins. **(A)** Yeast two-hybrid assays showing the interaction between BnMED16 and BnMED25. **(B)** Yeast two-hybrid assays showing the interaction between BnMED25 and BnMYC2, BnMED25 and BnCOI1, BnMED25, and BnEIN3. Transformed yeast cells were grown on SD media, and the colonies on TDO and QDO media indicate positive interactions. The positive control was pGADT7-RecT + pGBKT7-53 and the negative control was pGADT7-RecT + pGBKT7-lam. DDO, SD-Trp-Leu media; TDO, SD-Trp-Leu-His; QDO, SD-Trp-Leu-His-Ade; BnMED25-AD, pGADT7-BnMED25; BnMYC2-AD, pGADT7-BnMYC2; BnCOI1-AD, pGADT7-BnCOI1; BnEIN3-AD, pGADT7-BnEIN3; BnMED16-BD and BnMED25-BD, pGBKT7-BnMED16 and pGBKT7-BnMED25. **(C)** BiFC assay showing that the interaction between nYFP-BnMED16 and cYFP-BnMED25, nYFP-BnMED25, and cYFP-BnMYC2, nYFP-BnMED25 and cYFP-BnCOI1, nYFP-BnMED25 and cYFP-BnEIN3 formed a functional YFP in the nucleus. mCherrey served as a nucleus marker. Merge = merging of YFP and mCherrey. The interactions between nYFP-BnMED16 and cYFP, nYFP-BnMED25 and cYFP were used as negative controls for the BiFC assay. Bars, 50 μm. All the experiments were repeated at least three times with similar results.

To elucidate the molecular mechanism of BnMED16 for disease resistance, we explored its interacting proteins. Firstly, we subcloned the coding regions of BnMED16 into the yeast two-hybrid bait vector pGBKT7 (with the Gal4 binding domain) and transformed the resulting plasmid pGBKT7-BnMED16 (BnMED16-BD) into the yeast (*S. cerevisiae*) Y2HGold yeast strain. Meanwhile, we selected candidate proteins (BnMYC2, BnERF1, BnORA59, BnEIN3, BnCOI1, BnWRKY15, BnWRKY75, BnMED25, and BnMED14) based on previous reports and the RNA-seq results (Figure 3 and Supplementary Figures 4, 5) and qRT-PCR (Figures 2F, 3C and Supplementary Figure 6), subsequently subcloned their coding regions into the yeast two-hybrid the prey vector pGADT7 (with the Gal4 activation domain) and transformed the resulting plasmids into the yeast strain Y187. To confirm the interaction, Yeast two-hybrid (point-to-point) assay was performed between BnMED16-BD and the candidate proteins fused with the Gal4 activation domain. It revealed that only BnMED25 could interacted with BnMED16 among the candidate proteins (Figure 5A). In biomolecular fluorescence complementation (BiFC) assays, we found the co-transformation of MED16-nYFP and MED25-cYFP which produced a strong YFP signal in the nuclei of tobacco leaves (Figure 5C).

Since it has been reported that AtMED25 plays a role in JA/ET signaling by interacting with MYC2, COI1, and EIN3 in *Arabidopsis* (Çevik et al., 2012; Yang et al., 2014; An et al., 2017), we further investigated the interactions between BnMED25 and JA/ET signal related proteins. We observed that BnMED25 interacted with BnMYC2, BnCOI1, and BnEIN3 in yeast (Figure 5B) and nucleus *of N. benthamiana* leaves, respectively (Figure 5C). Taken together, these results suggested that BnMED16 may indirectly regulate JA/ET signal pathway by physically associated with BnMED25 to confer the *S. sclerotiorum* resistance.

### BnMED16 Is Physically Associated With BnWRKY33

**FIGURE 6:**
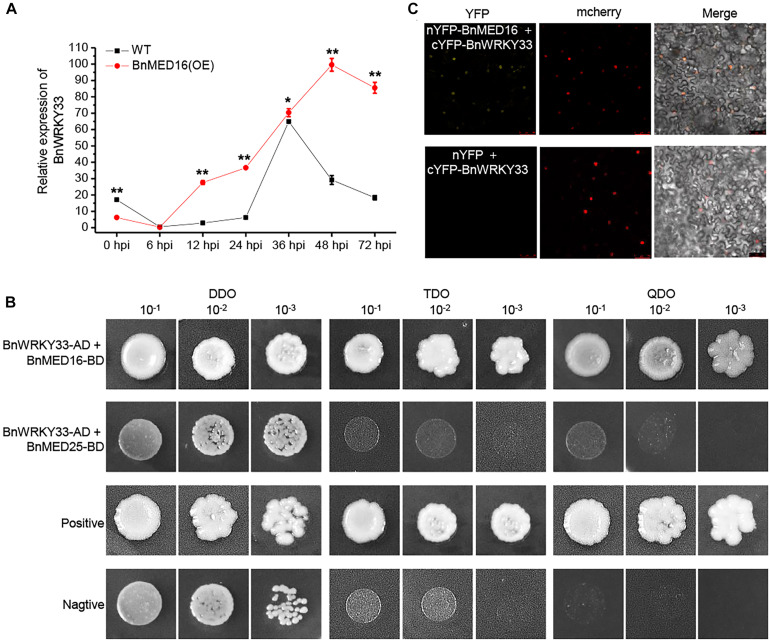
Physical association between BnMED16 and BnWRKY33. **(A)** Expression pattern of *BnWRKY33* (*BnaC04g06800D*) in BnMED16(OE) and WT seedling leaves at different time points after inoculation with *S. sclerotiorum*. *BnActin7* was used as the internal control; Data are means ± SD of three biological replicates and the asterisks denote statistical significance at *P* < 0.01 (**) or *P* < 0.05 (*) between the WT and BnMED16(OE) lines at each time point by Student’s *t*-tests. **(B)** Yeast two-hybrid assays showing the interaction between BnWRKY33 and BnMED16. Transformed yeast cells were grown on SD media, and the colonies on TDO and QDO media indicate positive interactions. The positive control was pGADT7-RecT + pGBKT7-53 and the negative control was pGADT7-RecT + pGBKT7-lam. DDO, SD-Trp-Leu media; TDO, SD-Trp-Leu-His; QDO, SD-Trp-Leu-His-Ade; BnWRKY33-AD, pGADT7-BnWRKY33; BnMED16-BD and BnMED25-BD, pGBKT7-BnMED16 and pGBKT7-BnMED25. **(C)** BiFC assay showing that the interaction between nYFP-BnMED16 and cYFP-BnWRKY33 formed a functional YFP in the nucleus. mCherrey served as a nucleus marker. Merge = merging of YFP and mCherrey. The interactions between nYFP and cYFP-BnWRKY33 were used as negative controls for the BiFC assay. The other two negative controls were shown in Figure 5C as they did at the same time. Bars, 50 μm. All the experiments were repeated at least three times with similar results.

Previous work has proven the AtWRKY33 could mediate resistance against the necrotrophic fungal pathogens in *Arabidopsis* (Zheng et al., 2006; Wang et al., 2015; Zhou et al., 2020) and BnWRKY33 may confer the response to *S. sclerotiorum* by enhancing the expression of genes involved in camalexin synthesis in *B napus* (Ren et al., 2008; Liu et al., 2018). Moreover, AtMED16 was found to interact with AtWRKY33 to mediated WRKY33-activated defense signaling (Wang et al., 2015). Here, we detected that the majority of DEGs involving the camalexin synthesis pathway were dramatically induced in *BnMED16* overexpressing lines (Supplementary Figure 5B). Therefore, we suspected that BnWRKY33-mediated SSR resistance might depend depends on BnMED16. To test this, we investigated the expression patterns of *BnWRKY33* genes in BnMED16(OE) and WT seedling leaves at 0, 6, 12, 24, 36, 48, and 72 hpi and found that its transcript level was much higher in BnMED16(OE) leaves after 6 hpi (Figure 6A), indicating that overexpression of *BnMED16* could also promote the expression level of *BnWRKY33*. We then confirmed the interaction between BnMED16 and BnWRKY33 by Yeast two-hybrid (point-to-point) assay in yeast (Figure 6B) and BiFC assay in the nucleus *of N. benthamiana* leaves (Figure 6C), while BnMED25 and BnWRKY33 did not interact with each other (Figure 6B). Overall, it is likely that BnWRKY33-mediated resistance against *S. sclerotiorum* depends on BnMED16.

## Discussion

### BnMED16 Is a Downstream Signaling Component of Basal Resistance Against *S. sclerotiorum* in *B. napus*

The mechanism of plant defense against the broad-host-range necrotrophy is very complex, and the dominant form responses to pathogens is quantitative resistance (Mengiste, 2012; Wu et al., 2019). A complex defense mechanism is activated when infected by pathogens in plants. Recognition of pathogen-associated molecular patterns (PAMPs) or damage-associated molecular patterns (DAMPs) triggers the plant immune response (PTI/DTI) and results in down-stream immune response syndrome (Mengiste, 2012; Bacete et al., 2018). It includes synthesis of reactive oxygen species (ROS), increases of intracellular calcium (Ca^2+^) concentration (Baxter et al., 2014; Seybold et al., 2014), secretion of defense proteins (Stotz et al., 2011), reinforcement of cell wall via polysaccharide deposition and lignification, and changes of plant endogenous hormone synthesis and transduction, and so on (Peltier et al., 2009; Robert-Seilaniantz et al., 2011; Chowdhury et al., 2014). Numerous studies have shown that PTI is the main source of quantitative resistance of rapeseed to *S. sclerotiorum* (Kou and Wang, 2010; Lai and Mengiste, 2013).

The plant mediator is a multi-subunit complex, which acts as a transcription cofactor and participates in plant disease resistance (Bäckström et al., 2007; Dhawan et al., 2009; Kidd et al., 2009; Mathur et al., 2011; Canet et al., 2012; Zhang et al., 2012, 2013; Caillaud et al., 2013; Zhu et al., 2014; Wang et al., 2015; Zhai and Li, 2019; Ren et al., 2020). The majority function analysis of plant mediators has been performed in *Arabidopsis*, but little is known about their roles and molecular mechanism in oil rapeseed. Our study revealed that the Ca^2+^ signal pathway, ROS and MAP kinases pathway related genes as well as RLKs and WAK genes were differentially expressed in *BnMED16* overexpressing lines at early stage of *S. sclerotiorum* infection (Figure 3, Supplementary Figure 5, and Supplementary Dataset 1), indicating the typical early reactions of PTI/DTI were enhanced in *BnMED16* overexpression plants, such as increased expression of membrane receptors of P/DAMP, an increase in intracellular Ca^2+^ concentrations, a burst of ROS, activation of MAP signaling cascade, etc. Our results also proved that overexpression of *BnMED16* gave the plants enormous power to activate various “soldiers” to limit further infection of *S. sclerotiorum*, enhancing the lignification of the plant cell wall to reinforce the resistance barrier (Figure 3), it includes improving the antimicrobial enzyme activities of β-1-3-glucanase and chitinase to degrade the cell wall of *S. sclerotiorum* to kill the pathogens (Supplementary Figure 3), and promoting the synthesize of PGIPs to inhibit the degradation of the plant cell wall by polygalacturonase secreted by *S. sclerotiorum* (Figure 3 and Supplementary Figure 6). All the reactions occurred in final stage of PTI (Williams et al., 2011), we therefore speculate that BnMED16 is a downstream signaling component and does not depend on PTI. However, it may augment PTI responses.

Although the roles of ROS in plants appeared to be more complex with *S. sclerotiorum* infection, it remains interesting to investigate how ROS affect plant disease resistance in *BnMED16* transgenic plants in the future. Also, it is worth studying the changes of plant cell wall composition before and after *S. sclerotiorum* inoculation, thus to figure out whether the resistance depend on DTI or not in *B. napus*. More importantly, previous studies on the MED family mainly focuses on the gene function analysis by using mutants in *Arabidopsis*, our study adopted the strategy of constitutively overexpressing *BnMED16* driving by double CaMV 35S promoter in *B. napus* to further explore whether its function of disease resistance is conserved or not in crop. Here, our results showed that increased expression of *BnMED16* enhanced *B. napus* field resistance to SSR (Figures 2D,E) without negative impact on plant growth (Supplementary Figure 2), which provides high resistant resources for SSR and benefit for breeding and cultivating of *Sclerotinia*-resistant rape varieties.

### BnMED16 Confers *S. sclerotiorum* Resistance by Enhancing BnMED25-Mediated JA/ET Defense Pathways

The tail module of the MED subunits could interact with specific TFs to regulate different biological processes (Chadick and Asturias, 2005; Casamassimi and Napoli, 2007; Tsai et al., 2014; Yang et al., 2016). Although topological positions of most mediators are still unclear, studies have indicated that the AtMED16 and AtMED25 located in the tail module since they could interact with many TFs in *Arabidopsis* (Zhu et al., 2011; Çevik et al., 2012; Yang et al., 2014; Wang et al., 2015, 2019; An et al., 2017; Guo et al., 2020). Here, it is worth noting that the expression of *BnMED25* was unchangeable in WT (Figure 1), but significantly induced by the enhanced transcript level of *BnMED16* upon *S. sclerotiorum* infection (Figure 3 and Supplementary Figure 6). We additionally confirmed that BnMED16 directly interacts with BnMED25 (Figure 5), which is consistent with previous studies in *Arabidopsis* that AtMED16 regulates iron homeostasis through AtMED25 (Yang et al., 2014) and AtMED16 could associate with AtMED25 to participate in the ABA response (Guo et al., 2020). Although it remains unclear how BnMED16 induced BnMED25, it is possible that increase of BnMED16 subunits change the conformation of the mediator complex so that the tail subunits including BnMED16 and BnMED25 are exposed to recruit proteins involved in the resistance to SSR, or perhaps that BnMED16 recruits certain specific factors, which are required for the activation of BnMED25.

It is well known that JA/ET has a synergistic effect against necrotrophic fungal pathogens (Kunkel and Brooks, 2002; Glazebrook, 2005). They cooperate to induce the pathogen-defense gene PDF1.2, which regulated by APETALA2/ETHYLENE-RESPONSIVE FACTORs (AP2/ERFs) domain TFs, OCTADECANOID-RESPONSIVE ARABIDOPSIS ETHYLENE/ETHYLENE-RESPONSIVE FACTOR59 (ORA59) and ERF1 (Pré et al., 2008). Noteworthy, genes encoding the AP2/ERF factors are controlled by two TFs, EIN3 and ETHYLENE INSENSITIVE3-LIKE1 (EIL1) and these two TFs not only regulates ET response but also corporates in the signal center of JA/ET (An et al., 2010; Zhu et al., 2011). Meanwhile, numerous studies proved that AtMED25 plays crucial roles in the JA/ET signaling pathway in *Arabidopsis* by interacting with some key TFs like AtMYC2, AtJAZ1, AtCOI1, AtEIN3/AtEIL1, AtORA59, and AtERF1 (Çevik et al., 2012; Yang et al., 2014; An et al., 2017). We thus further analyzed the correlation between the two tail subunits (BnMED16 and BnMED25) and the JA/ET signal pathway associated TFs. Interestingly, BnMED25 but not BnMED16 interacts with JA/ET defense pathways-associated TFs BnMYC2, BnCOI1, and BnEIN3 (Figure 5). Considered previous reports have been proven JA/ET has a synergistic effect against necrotrophic fungal pathogens (Kunkel and Brooks, 2002; Glazebrook, 2005), and our data showed the majority genes involved in JA/ET synthesis and transduction pathway rather than that in SA were notably induced by the overexpression of *BnMED16* (Figure 3 and Supplementary Figure 6). Thus, it is reasonable to assume that BnMED16 confers *S. sclerotiorum* resistance by enhancing BnMED25-mediated JA/ET defense pathways. Indeed, it is worth further study how BnMED16 associates with BnMED25 to participate in the *B. napus* SSR resistance. Perhaps BnMED16 or BnMED25 knock-out lines would be more useful, but it is difficult to obtain the homozygous and this part of the experiment is still in progress.

### BnMED16 Confers *S. sclerotiorum* Resistance by Mediating BnWRKY33-Activated Defense Signaling

The phytoalexin camalexin plays an important role in plant responses to a variety of pathogens (Ren et al., 2008; Stotz et al., 2011; Wang et al., 2015; Liu et al., 2018; Zhou et al., 2020). It has been well characterized that the WRKY33 plays a pivotal role by probably binding to the promoters of CYP71A13 and PAD3 *in vivo* (Zheng et al., 2006). Additionally, WRKY33 functions as the substrate of both pathogen-responsive CALCIUM-DEPENDENT PROTEIN KINASE5/6 (CPK5/CPK6) and MPK3/MPK6, which cooperatively regulate camalexin biosynthesis by differentially phosphorylating of WRKY33 in *Arabidopsis* (Ren et al., 2008; Zhou et al., 2020). Here, the elevated expression of *BnWRKY33* was detected in *BnMED16* overexpressing lines and the interaction between BnWRKY33 and BnMED16 was observed in yeast and nucleus of *N. benthamiana* leaves (Figure 6). Moreover, a group of camalexin synthesis related genes like *BnCPK6*, *BnMPK6*, *GSTs*, *CYP79B2*, *CYP71A13*, *CYP71B7*, *CYP71B28*, and *PAD4* were differentially expressed in *BnMED16* overexpressing lines (Figure 3 and Supplementary Figure 5B). A recent study indicated that BnWRKY33 can also affect the expression of genes regulated by SA and JA (Liu et al., 2018). Therefore, the resistance-inducing properties of BnWRKY33 may include both the synthesis of camalexin and the activation of JA or SA signal pathways. Given these findings, it may be concluded that BnMED16 confers *S. sclerotiorum* resistance also by inducing BnWRKY33-activated defense signaling.

## Conclusion

**FIGURE 7:**
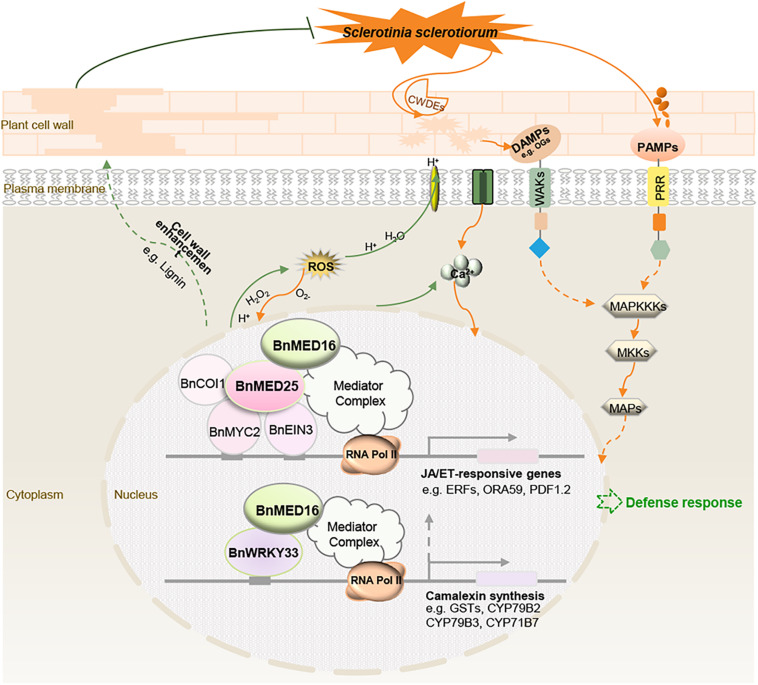
Schematic model illustrating the proposed role of BnMED16 confers *S. sclerotiorum* resistance through increased activation of defense mechanisms in *B. napus*. BnMED16 positively regulates plant defense against *S. sclerotiorum* probably via BnMED25-mediated JA/ET defense pathways and BnWRKY33-activated defense signaling. Overexpression of BnMED16 results in the enhanced basal immunity in *B. napus* by promoting the accumulation and clearance of ROS upon *S. sclerotiorum* infection, increasing the expression of PR and phytoalexins genes, enhancing the cell wall reinforcement and cell wall-mediated resistance by lignification and pectins deposition, and inducing plant camalexin synthesis, and so on. CWDEs, cell wall-degrading enzymes; DAMPs, damage-associated molecular patterns; PAMPs, pathogen-associated molecular patterns; PRR, pattern recognition receptors; ROS, reactive oxygen species; MAPs, mitogen-activated proteins; MAPKKKs, MAP-kinase-kinase kinases; MKKs, MAP-kinase kinases; OGs, oligogalacturonides.

We propose a hypothetical model for BnMED16-conferred resistance to *S. sclerotiorum* in *B. napus* (Figure 7). BnMED16 functions as a key component of basal resistance against *S. sclerotiorum*, which possibly affects the recognition of P/DAMP, the concentrations of intracellular Ca^2+^, a burst of ROS, the activation of MAP signaling cascade and RLKs, the lignification of the plant cell, the synthesis of phytoalexin camalexin, and so on. The most likely mechanism is that BnMED16 positively regulates plant defense against *S. sclerotiorum* via BnMED25-mediated JA/ET defense pathways and BnWRKY33-activated defense signaling.

## Data Availability Statement

The original contributions presented in the study are publicly available. This data can be found at NCBI (https://www.ncbi.nlm.nih.gov/): BioProject, PRJNA744325; BioSamples, SAMN20087435; SRA: SRR15058739, SRR15058740, SRR15058741, SRR15058742, SRR15058743, and SRR15058744.

## Author Contributions

HH, YT, and JW completed major experiments, and wrote the manuscript. FC and YY contributed to the plant growth and *S. sclerotiorum* inoculation. XP participated in vector construction and transformation. XDo and XJ participated in transgenic lines identification. SL co-supervised experiments. XDu designed the project, interpreted data and finalized the manuscript. All authors approved the final manuscript.

## Conflict of Interest

The authors declare that the research was conducted in the absence of any commercial or financial relationships that could be construed as a potential conflict of interest. The handling Editor declared a past co-authorship with one of the authors JW.

## Publisher’s Note

All claims expressed in this article are solely those of the authors and do not necessarily represent those of their affiliated organizations, or those of the publisher, the editors and the reviewers. Any product that may be evaluated in this article, or claim that may be made by its manufacturer, is not guaranteed or endorsed by the publisher.

## Footnotes

1
https://imagej.nih.gov/ij/

2
http://brassicadb.org/brad/datasets/pub/BrassicaceaeGenome/Brassica_napus/Brassica_napus.annotation_v5.gff3.gz

3
http://www.broadinstitute.org/annotation/genome/sclerotinia_sclerotiorum/MultiHome.html
