# Supplementary material for: Brassica napus Mediator Subunit16 Induces BnMED25- and BnWRKY33-Activated Defense Signaling to Confer Sclerotinia sclerotiorum Resistance
Source: Front Plant Sci. 2021 Aug 19;12:663536. doi: 10.3389/fpls.2021.663536 (PMC8417128; doi:10.3389/fpls.2021.663536)
Supplement: Supplementary file 2 [file Data_Sheet_2.docx]

**Supporting Information**

**Article Title: *Brassica napus* mediator subunit16 induces BnMED25- and BnWRKY33-activated defense signaling to confer *Sclerotinia sclerotiorum* resistance**

­­

*Submitted to Plant Breeding, a section of the journal Frontiers in Plant Science*

Author names: Huizhen Hu, Yiwei Tang, Jian Wu, Feizhi Chen, Yidan Yang, Xuancheng Pan, Xiang Dong, Xianda Jin, Sheng Liu and Xuezhu Du

Affiliation: State Key Laboratory of Biocatalysis & Enzyme Engineering, Hubei Collaborative Innovation Center for Green Transformation of Bio-resources, Hubei Key Laboratory of Industrial Biotechnology, College of Life Science, Hubei University, Wuhan 430062, Hubei, China; Southwest Research Center for Landscape Architecture Engineering, State Forestry and Grassland Administration, Yunnan Province Engineering Research Center for Functional Flower Resources and Industralization, College of Landscape Architecture and Horticulture Sciences, Southwest Forestry University, Kunming 650224, Yunnan, China; Key Laboratory of Plant Functional Genomics of the Ministry of Education, Yangzhou University, Yangzhou 225009, Jiangsu, China; Key Laboratory of Biology and Genetic Improvement of Oil Crops, Ministry of Agriculture and Rural Affairs, P.R. China, Wuhan 430062, Hubei, China

* To whom correspondence should be addressed.

E-mail: duxuezhusk@163.com

**Supplementary Table 1** Primers used in this study

| **Purpose** | **Genes/Vectors** | **Primer name** | **Primer sequence (5’-3’)** | **Restriction Enzyme** | **Length of amplification (bp)** |
| --- | --- | --- | --- | --- | --- |
|  |  |  |  |  |  |
| qRT-PCR | *BnMED16* | Q-BnMED16-F | CTTAAACAGCCCAACTCCAATC | / | 228 |
|  | BnaA09g20140D | Q-BnMED16-R | AGAGTCTGCAACAACATTGAAC |  |  |
|  | *BnMED14* | Q-BnMED14-F | CAGCGACTTCAAGACAATCAAA | / | 219 |
|  | BnaAnng10600D | Q-BnMED14-R | CACGGAGTACTACAGAGACATC |  |  |
|  | *BnMED25* | Q-BnMED25-F | CTCCAAAGGACTTGCGATTATG | / | 160 |
|  | BnaA09g28640D | Q-BnMED25-R | GTTCCTCTCTCCGTTTAACTCA |  |  |
|  | *BnMED8* | Q-BnMED8-F | ATCAAATGCCATATTCGCAGTC | / | 208 |
|  | BnaA09g53670D | Q-BnMED8-R | GACATGTTTGGTATCATCTGGC |  |  |
|  | *BnMED21* | Q-BnMED21-F | TTGTCGGAGAATCAGTAGATCG | / | 158 |
|  | BnaA09g20250D | Q-BnMED21-R | CGTATTGCGAAATCCCAGTAGA |  |  |
|  | *BnMED19a* | Q-BnMED19a-F | GCGTGATAAAGAAGATGGAACC | / | 147 |
|  | BnaC09g44480D | Q-BnMED19a-R | AATCTGATAGAGACGCGGTAAG |  |  |
|  | *BnWRKY33* | Q-BnWRKKY33-F | GTACTTTCCCAAACTGTCCAAC | / | 131 |
|  | BnaC04g06800D | Q-BnWRKKY33-R | CGAAGAAGACGACGATCTTCTA |  |  |
|  | *BnPR1*  BnaC01g04530D | Q-BnPR1-F | ATCTCCGAAAAGGCCAGGGA | / | 118 |
|  |  | Q-BnPR1-R | TACCATTTACCGCATCGCCC |  |  |
|  | *BnPR2* | Q-Bn13EG-F | GTAACGAAGTTGTACCGTCCAACG | / | 127 |
|  | BnaA01g17540D | Q-Bn13EG-R | CAAAGTCATATCAACCGCTGTAGAC |  |  |
|  | *BnPR3*  *BnaA05g26640D* | Q-BnChitinase-F | GGTAACACCGAACCTTACTGTA | / | 194 |
|  |  | Q-BnChitinase-R | GACTTAGCGGCATTGATAAAGG |  |  |
|  | *BnPDF1.2*  BnaC02g23620D | Q-BnPDF1.2-F | GCATGAATCAGTGCATTAACCT | / | 173 |
|  |  | Q-BnPDF1.2-R | AATCCACACACAATTAAGCACC |  |  |
|  | *BnJAZ1* | Q-BnJAZ1-F | TACGGCGGGCAAGTGATT | / | 175 |
|  | BnaA06g13250D | Q-BnJAZ1-R | TTGGTTCGGGGTAGGAGC |  |  |
|  | *BnMYC2* | Q-BnMYC2-F | TTATGGGTCGGATCAGTTAACC | / | 152 |
|  | BnaC05g28450D | Q-BnMYC2-R | TCATAAGATCCGAACTCTGTCG |  |  |
|  | *BnCOI1* | Q-BnCOI1-F | GCATTCTATCTCAGACAAAGCG | / | 166 |
|  | BnaA03g56600D | Q-BnCOI1-R | CTCTCATCTCCAGCTTCTGTAG |  |  |
|  | *BnEIN3* | Q-BnEIN3-F | GAGGATCTGGGATAACTGTGAG | / | 201 |
|  | BnaA05g20160D | Q-BnEIN3-R | CATCCATCGTTCCTACTACTCC |  |  |
|  | *BnORA59*  BnaC08g44670D | Q-BnORA59-F | TCATACAGAGGAGTGAGGAAGA | / | 242 |
|  |  | Q-BnORA59-R | GGAGACTCTCCGTTATCGAAAT |  |  |
|  | *BnERF1*  BnaA01g23940D | Q-BnERF1-F | ATGCTTCTCTACGGACTAATCG | / | 271 |
|  |  | Q-BnERF1-R | CCGGAAAATTTAATATCGCCGA |  |  |
|  | *BnPGIP1*  BnaC09g48690D | Q-BnPGIP1-F | TCATTTGGATCATTCCCAGGAA | / | 123 |
|  |  | Q-BnPGIP1-R | CTTGTTCCGTGAGAAATCGATC |  |  |
|  | *BnCYP71B7*  BnaC08g40600D | Q-BnPAD3-F | TCGCGTTTGGGATAGATATTCA | / | 162 |
|  |  | Q-BnPAD3-R | GTGTCTTGTTCTGTCCTGAAAC |  |  |
|  | *BnCSD1* | Q-BnCSD1-F | TCGATCATTGACATGTTTGCTC | / | 126 |
|  | BnaA06g05150D | Q-BnCSD1-R | AGACACAACCGGATATGAGATC |  |  |
|  | *BnFSD2*  BnaA03g13310D | Q-BnFSD2-F | GGTCTCATCTGACTAGGTAAGC | / | 211 |
|  |  | Q-BnFSD2-R | GCTCAGACTTTTATTAGCGCAA |  |  |
|  | *BnCAT2* | Q-BnCAT2-F | CAAAGCTCACTACGTGAAGTTC | / | 206 |
|  | BnaA03g53180D | Q-BnCAT2-R | GGTCAAAGTCGAACTTGTCTTC |  |  |
|  | *BnCAT3* | Q-BnCAT3-F | CACGCAACTAAAGATCTCCATG | / | 131 |
|  | BnaA08g21730D | Q-BnCAT3-R | CATATCTTGGTCACATCAAGCG |  |  |
|  | *BnPER21* | Q-BnPER21-F | TTGCCCAAGTCCAAACCC | / |  |
|  | BnaC03g20530D | Q-BnPER21-R | GACCAGGAGCCCTCTATG |  |  |
|  | *BnPRX34* | Q-BnPRX34-F | CATCGTACGAGAAACCATTGTC | / | 252 |
|  | BnaA01g20660D | Q-BnPRX34-R | ATATCTGCGCATGAAACAGTTC |  |  |
|  | *BnGPX7* | Q-BnGPX7-F | ACATAGTATCGACCACGTGTTT | / | 191 |
|  | BnaA03g51760D | Q-BnGPX7-R | TGTCCGTATGTAGAACACAAGT |  |  |
|  | *BnAPX1*  BnaA09g49190D | Q-BnAPX1-F | TGAAATCTTCTGTTGGTTGCAG | / | 134 |
|  |  | Q-BnAPX1-R | ACCGCCTAAAAGAACACAAATC |  |  |
|  | *BnActin7* | Q-BnActin7F1 | TCTTCCTCACGCTATCCTCCG | / | 181 |
|  |  | Q-BnActin7R1 | AGCCGTCTCCAGCTCTTGC |  |  |
| OE vector | *pCAMBLA1300-2301-D35S:BnMED16* | BnMED16-F | CGGAATTCTTGCTCTCTCGCTCGACAACGATCAG | EcoR I | 3741 |
|  |  | BnMED16-R | ACGCGTCGACATCCCACGCAATTTTGTCATCAAAC | SalI |  |
| Y_2_H vector | *pGBKT7-BnMED16* | BD-BnMED16-F | GAATTCATGAATCAGCCCCAAGTTTCTC | EcoRI | 3744 |
|  |  | BD-BnMED16-R | GGATCCCTATACAACACGGACCCACGTT | BamHI |  |
|  | *pGBKT7-BnMED25* | BD-BnMED25-F | GAATTCATGTCGTCGGAGTTGAAACAG | EcoRI | 2466 |
|  |  | BD-BnMED25-R | GGATCCTTATCCCATGAAGCCCGC | BamHI |  |
|  | *pGADT7-BnMED25* | AD-BnMED25-F | GAATTCATGTCGTCGGAGTTGAAACAG | EcoRI | 2466 |
|  |  | AD-BnMED25-R | GGATCCTTATCCCATGAAGCCCGC | BamHI |  |
|  | *pGADT7-BnMYC2* | AD-BnMYC2-F | GAATTCATGACGGAGCCGACGATG | EcoRI | 1827 |
|  |  | AD-BnMYC2-R | GGATCCTTAACCAATCTTTGAGATTAAACTCG | BamHI |  |
|  | *pGADT7-BnCOI1* | AD-BnCOI1-F | GAATTCATGGAGGATCCAGACATCAAGA | EcoRI | 1809 |
|  |  | AD-BnCOI1-R | GGATCCTCATGTCTCCTCCTTTAGTACTTTAAC | BamHI |  |
|  | *pGADT7-BnERF1* | AD-BnERF1-F | GAATTCATGGATCCATATTTTGTTCAGTCA | EcoRI | 633 |
|  |  | AD-BnERF1-R | GGATCCTCACCAGGCCCCACTGTT | BamHI |  |
|  | *pGADT7-BnORA59* | AD-BnORA59-F | GAATTCATGGAGTTTCAAACTAACTTCTTTTCT | EcoRI | 735 |
|  |  | AD-BnORA59-R | GGATCCTCATGAACATAATCTCATAAGCTCTTC' | BamHI |  |
|  | *pGADT7-BnEIN3* | AD-BnEIN3-F | GAATTCATGATGTTTAACGAGATGGGAATG | EcoRI | 1881 |
|  |  | AD-BnEIN3-R | GGATCCTTAGAACCATATGGATACATCTTGCT | BamHI |  |
|  | *pGADT7-BnWRKY33* | AD-BnWRKY33-F | GAATTCATGGCTGCTTCTTCCCTCC | EcoRI | 1473 |
|  |  | AD-BnWRKY33-R | GGATCCTCACGACAAGAACGAATCAAAA | BamHI |  |
|  | *pGADT7-BnMED14* | AD-BnMED14-F | GAATTCATGGCGGAATTAGGGCAAC | EcoRI | 4893 |
|  |  | AD-BnMED14-R | GGATCCCTATATGGTAAACTCCTTTTGAAGAGTG | BamHI |  |
|  | *pGADT7-BnWRKY15* | AD-BnWRKY15-F | GAATTCATGGCGGTGGAGCTCATGAC | EcoRI | 897 |
|  |  | AD-BnWRKY15-R | GGATCCTCAAGACGATTCCAAAATGAGAT | BamHI |  |
|  | *pGADT7-BnWRKY75* | AD-BnWRKY75-F | GAATTCATGGAGGGATATCAAAATGGATC | EcoRI | 444 |
|  |  | AD-BnWRKY75-R | GGATCCTTAATTAAAAGAAGAGTAGATTTGCATT | BamHI |  |
| BIFC vector | *35s-BnMED16-Myc-nYFP* | BnMED16-nYFP-F | TCTAGAATGAATCAGCCCCAAGTTTCTC | XbaI | 3744 |
|  |  | BnMED16-nYFP-R | GGATCCTACAACACGGACCCACGTT | BamHI |  |
|  | *35s-BnMED25-Myc-nYFP* | BnMED25-nYFP-F | TCTAGAATGTCGTCGGAGTTGAAACAG | XbaI | 2466 |
|  |  | BnMED25-nYFP-R | GGATCCTCCCATGAAGCCCGC | BamHI |  |
|  | *35s-BnMED25-HA-cYFP* | BnMED25-cYFP-F | TCTAGAATGTCGTCGGAGTTGAAACAG | XbaI | 2466 |
|  |  | BnMED25-cYFP-R | GGTACCTCCCATGAAGCCCGC | KpnI |  |
|  | *35s-BnWRKY33-HA-cYFP* | BnWRKY33-cYFP-F | TCTAGAATGGCTGCTTCTTCCCTCC | XbaI | 1473 |
|  |  | BnWRKY33-cYFP-R | GGTACCCGACAAGAACGAATCAAAA | KpnI |  |
|  | *35s-BnMYC2-HA-cYFP* | BnMYC2-cYFP-F | TCTAGAATGACGGAGCCGACGATG | XbaI | 1827 |
|  |  | BnMYC2-cYFP-R | GGTACCACCAATCTTTGAGATTAAACTCG | KpnI |  |
|  | *35s-BnCOI1-HA-cYFP* | BnCOI1-cYFP-F | TCTAGAATGGAGGATCCAGACATCAAGA | XbaI | 1809 |
|  |  | BnCOI1-cYFP-R | GGTACCTGTCTCCTCCTTTAGTACTTTAAC | KpnI |  |
|  | *35s-BnEIN3-HA-cYFP* | BnEIN3-cYFP-F | TCTAGAATGATGTTTAACGAGATGGGAATG | XbaI | 1881 |
|  |  | BnEIN3-cYFP-R | GGTACCGAACCATATGGATACATCTTGCT | KpnI |  |

**
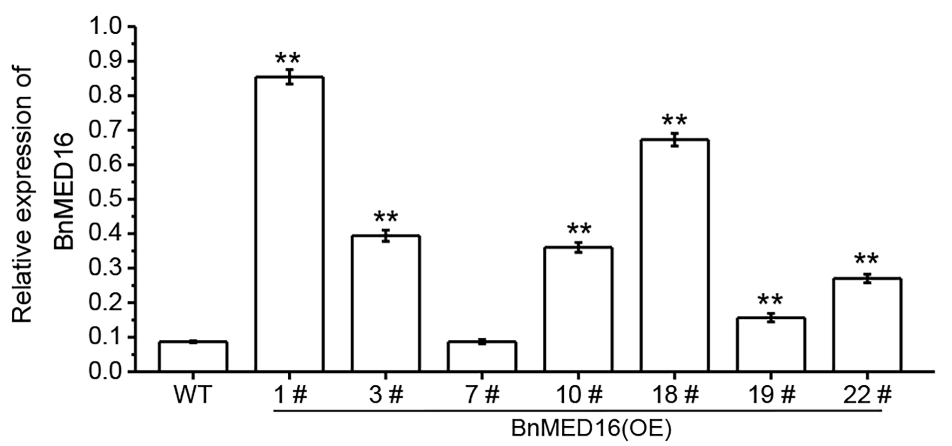
**

**Supplementary Figure S1** The expression level of *BnMED16* gene in T0 transgenic lines and wild type. WT, wild type; BnMED16(OE) was the transgenic plants that overexpressed *BnMED16* genes in *B. napus*; 1#, 3#, 7#, 10#, 18#, 19#, 22# indicates the individual T0 transgenic lines. *BnActin7* was used as the internal control. The data are the mean ± SD of three independent biological replicates and the asterisks denote statistical significance at *P* < 0.01 (**) between the WT and BnMED16(OE) lines by Student’s t-tests.

**
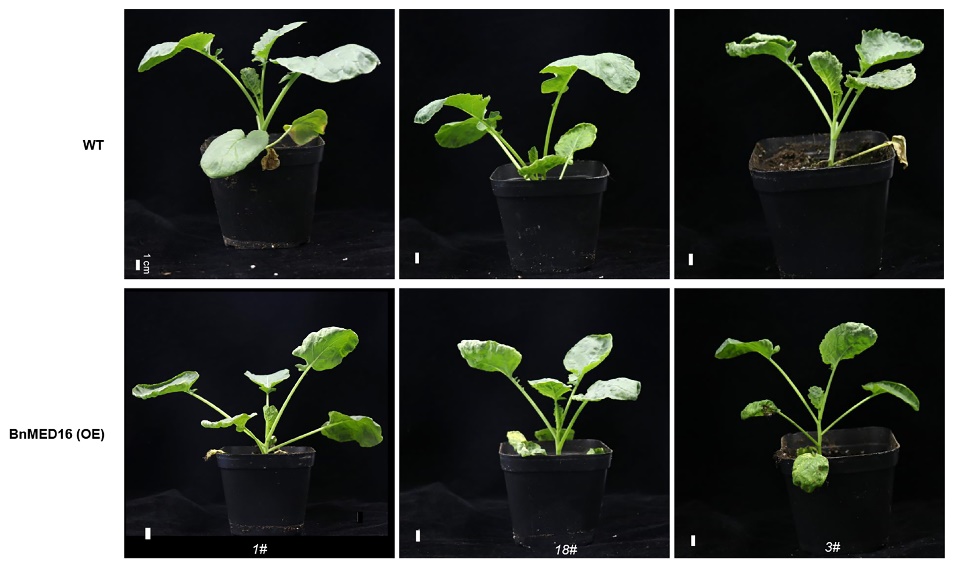
**

**Supplementary Figure S2** Phenotype of T4 homozygous BnMED16(OE) lines and WT seedlings. Bars, 1 cm.

**
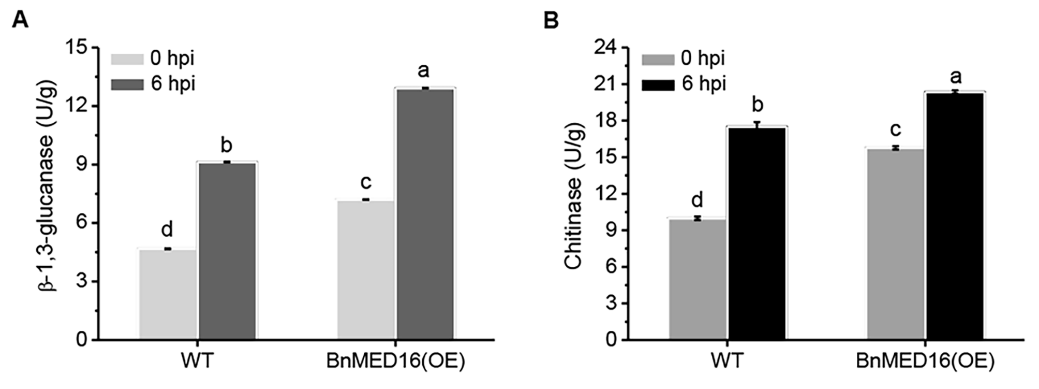
**

**Supplementary Figure S3** Analysis of defense protease activities of WT and *BnMED16* overexpression seedling leaves before and after inoculation with *S. sclerotiorum.* **(A)** Determination of β-1,3-glucanaseactivities; **(B)** Determination of chitinase activities. The data are the mean ± SD of three independent biological replicates. Least-significant difference (LSD) tests were used for multiple comparisons. Different letters above bars indicate that the means differ according to ANOVA and LSD tests (*P* < 0.01).

**
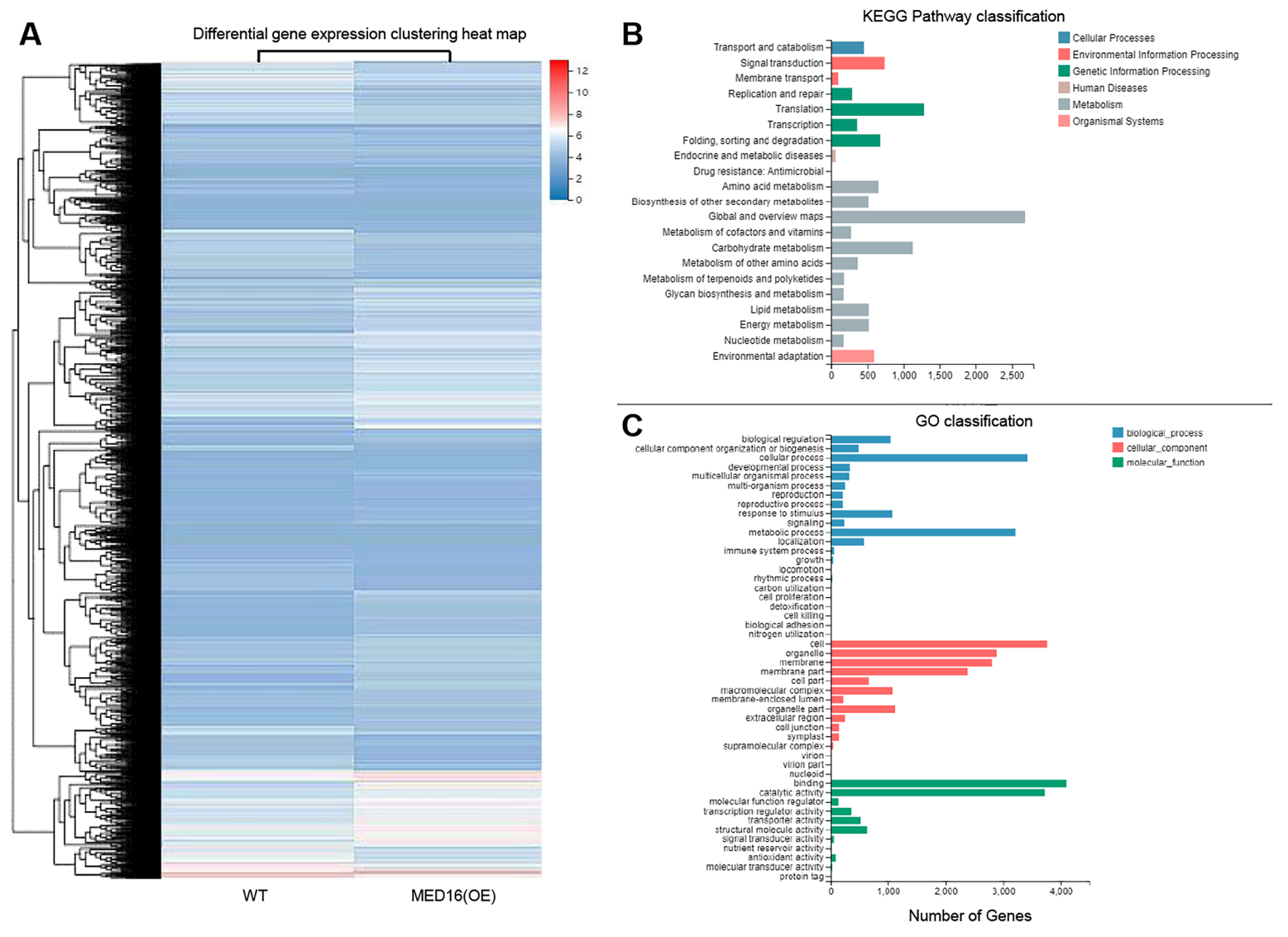
**

**Supplementary Figure S4** Clustered heatmap and signaling pathway enrichment analysis of differentially expressed genes (DEGs) of *B. napus* genes responsive to *S. sclerotiorum* between BnMED16 overexpression and WT lines. **(A)** All DEGs were clustered in the heatmap of based on FPKM (fragments per kilobase of exon per million fragments mapped) levels in BnMED16(OE) and WT seedling leaves in response to *S. sclerotiorum* infection after 6 hours. The DEGs are clustered on the Y-axis according to hierarchical agglomerative clustering. The FPKM was normalized using the *log_10_ (fpkm+1)* to generate the heatmap. Red and blue represent high and low gene expression, respectively; **(B,C)** KEGG pathway and gene ontology (GO) classification of DEGs as shown in **(A)**.

**
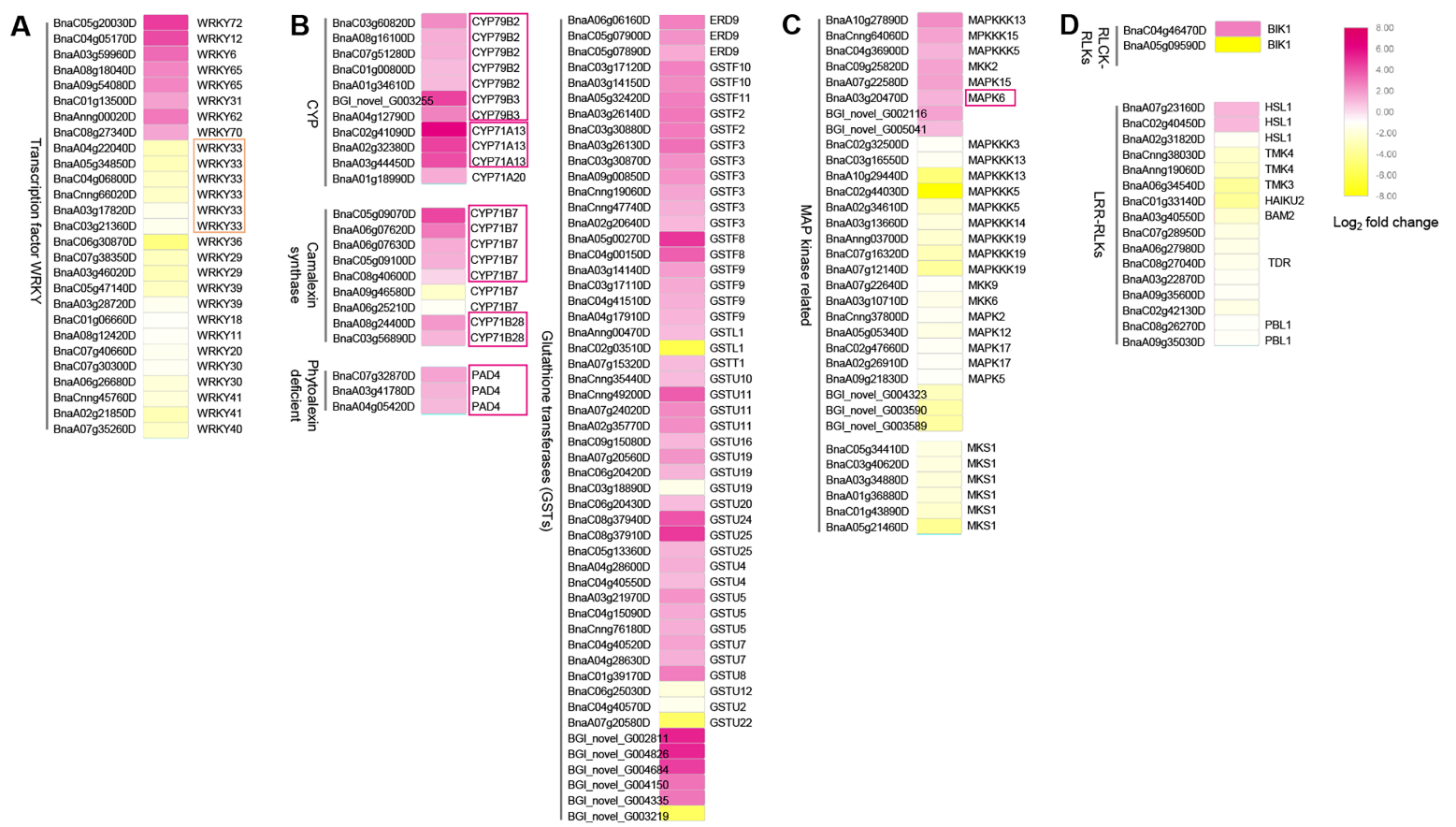
**

**Supplementary Figure S5** DEGs between transgenic and WT *B. napus* lines in response to *S. sclerotiorum* infection. Heatmap of DEGs related to *S. sclerotiorum* infection *B. napus* seedling leaves of transcription factor WRKY genes **(A)**, camalexin synthesis genes **(B)**, MAP kinases pathway genes **(C)**, and receptor-like protein kinases (RLKs) related genes**(D)**. DEGs were considered statistically significant if *q*-value <0.005 and |log_2_-fold change| >1. The log_2_-fold change is indicated according to the scale bar. The color from yellow to magenta represents the DEGs from down-regulated to up-regulated. The intensity of the color indicates the multiple of change for DEGs, with a darker color meaning higher fold change. The common up-regulated DEGs are highlighted by red boxes. Details of the genes are given in **Supplementary Dataset 1**.


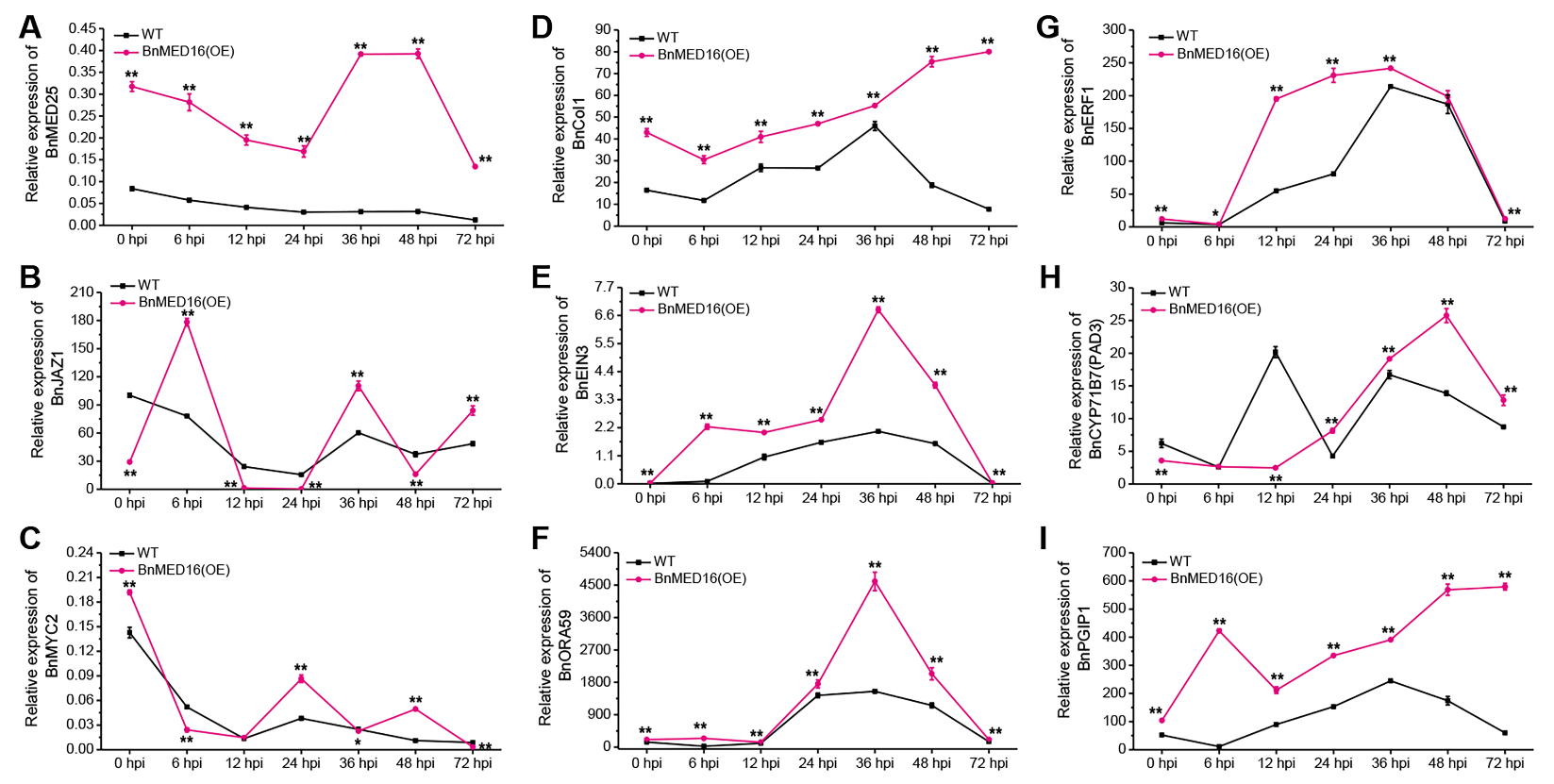


**Supplementary Figure S6** qRT-PCR confirmation of the DEGs between BnMED16 overexpression and WT lines after inoculation. Expression pattern of *BnMED25* (*BnaA09g28640D*) **(A)**, *BnJAZ1* (*BnaA06g13250D*) **(B)**, *BnMYC2* (*BnaC05g28450D*) **(C)**, *BnCOI1* (*BnaA03g56600D*) **(D)**, *BnEIN3* (*BnaA05g20160D*) **(E)**, *BnORA59* (*BnaC08g44670D*) **(F)**, *BnERF1* (*BnaA01g23940D*) **(G)**, *BnPAD3* (*BnaC08g40600D*) **(H)**, and *BnPGIP1* (*BnaC09g48690D*) **(I)** in BnMED16(OE) and WT seedling leaves at different time points after inoculation with *S. sclerotiorum*. *BnActin7* was used as the internal control; Data are means ± SD of three biological replicates and the asterisks denote statistical significance at *P* < 0.01 (**) or *P* < 0.05 (*) between the WT and BnMED16(OE) lines at each time point by Student’s t-tests.
